# Neuroprotective effects of *Liriope platyphylla* extract against hydrogen peroxide-induced cytotoxicity in human neuroblastoma SH-SY5Y cells

**DOI:** 10.1186/s12906-015-0679-3

**Published:** 2015-06-09

**Authors:** Hee Ra Park, Heeeun Lee, Hwayong Park, Jong Wook Jeon, Won-Kyung Cho, Jin Yeul Ma

**Affiliations:** Korean Medicine (KM)-Application Center, Korea Institute of Oriental Medicine (KIOM), 70, Cheomdan-ro, Dong-gu Daegu, 701-300 South Korea

**Keywords:** *Liriope platyphylla*, Neuroprotective effects, Antioxidant activity, Antiapoptotic effect

## Abstract

**Background:**

Oxidative stress is involved in neuronal cell death and mitochondrial dysfunction in neurodegenerative diseases. *Liriope platyphylla* (LP) has been suggested to have anti-inflammation, anti-bacterial, and anti-cancer effects. However, whether LP exerts neuroprotective effects on neuronal cells is unknown.

**Methods:**

The present study was performed to investigate the neuroprotective effects of LP extract (LPE) against hydrogen peroxide (H_2_O_2_)-induced injury in human neuroblastoma cells SH-SY5Y. To test neuroprotective effects of LPE, we performed cell viability assay, flow cytometry analysis and western blot analysis. In addition, mitochondrial membrane potential (MMP) and oxidative stress were performed to evaluate the anti-apoptotic and anti-oxidant effects.

**Results:**

LPE pretreatment conferred significant protection against the H_2_O_2_-induced decrease of SH-SY5Y cell viability. H_2_O_2_-induced increases of intracellular oxidative stress and mitochondrial dysfunction were attenuated by LPE pretreatment. Therefore, LPE pretreatment prevented SH-SY5Y cell injury. Treatment with H_2_O_2_ significantly induced poly(ADP ribose) polymerase (PARP) and caspase-3 cleavage, which was blocked by LPE. We found that p38 activation was involved in the neuroprotective effects of LPE.

**Conclusions:**

Current findings suggest that LPE exerts neuroprotective effects against H_2_O_2_-induced apoptotic cell death by modulating p38 activation in SH-SY5Y cells. Therefore, LPE has potential anti-apoptotic effects that may be neuroprotective in neurodegenerative diseases and aging-related dementia.

## Background

Oxidative stress is involved in neuronal cell death, which is one of the major causes of neurodegenerative diseases, including Alzheimer’s disease, Parkinson’s disease, and amyotrophic lateral sclerosis [1–4]. Some reactive oxygen species (ROS) are spontaneously generated, such as superoxide, peroxide, and hydroxyl radical, while ROS can also be generated due to exogenous factors, such as radiation or drug exposure [5–7]. ROS are generally found in the course of apoptotic cell death caused by intracellular microenvironmental changes. In this context, inhibiting ROS generation can be a useful way to protect normal neuronal cells from damage or death that lead to neurodegenerative diseases and aging-related cognitive decline [8–11].

*Liriope platyphylla* (LP) is a traditional herbal medicine, the roots of which have been widely used to brain-associated diseases such as forgetfulness and palsy in Donguibogam. Previous studies suggested that LP and its active compounds may exert beneficial effects in cases of viral infection, inflammation, asthma, diabetes, and obesity by modulating the mitogen-activated protein kinase (MAPK)/nuclear translocation of nuclear factor-κB (NF-κB) signaling pathway, as well as inflammatory proteins [12–16]. LP and red LP extract were reported to decrease amyloid-beta (Aβ_1–42_) peptide levels in the brain and increase nerve growth factor (NGF) levels in the serum of NSE/hAPP_swe_ transgenic mice and Tg2576 mice respectively [17, 18]. However, the anti-apoptotic and neuroprotective effects of LP against hydrogen peroxide (H_2_O_2_)-induced neuronal cell loss have not been studied.

Therefore, the present study was performed to investigate whether LP extract (LPE) has neuroprotective effects against H_2_O_2_-induced neuronal cell loss in SH-SY5Y neuroblastoma cells. We examined LPE-induced anti-apoptotic and anti-inflammatory effects, as well as the related signaling pathways.

## Methods

### Preparation of *Liriope platyphylla* Extracts (LPE)

Dried roots of LP were purchased from local vendor Hyundai Herbal Market (Yeongcheon, Korea) and deposited in the herbal bank of KM-Application Center, Korea Institute of Oriental Medicine (KIOM; Daejeon, Korea) after verifying by Professor Ki Hwan Bae of the College of Pharmacy, Chungnam National University (Daejeon, Korea). Ethanolic extract of LP was extracted in 70 % ethanol (50 g/390 ml) at 40 °C in shaking incubator for 24 h. After extraction, the solution was filtered through filter paper (Whatman filter paper #1), and then the filtrate was lyophilized (yield; 65.9303 %). The freeze-dried LPE powder (100 mg) was then dissolved in 1 ml 50 % DMSO (v/v) and filtered through a 0.22 μm syringe filter.

### Cell culture

SH-SY5Y cells (kindly provided by Prof. Jaewon Lee, Pusan National University, Korea) are human neuroblastoma-derived cell line and had neuron-like characteristic. These cells can differentiate into the neurons by induction of retinoic acid (RA). SH-SY5Y cells were cultured in a humidified 5 % CO_2_ incubator at 37 °C with RPMI 1640 media (Lonza, Walkersville, MD, USA) supplemented with heat inactivated 10 % fetal bovine serum (HyClone Laboratories, Utah, USA), 2 mM glutamine, and 1 % penicillin/streptomycin antibiotic mixture (Corning Incorporated, NY, USA).

### Cell viability analysis

Cell viability was evaluated by Cell Counting Kit-8 (CCK) assay (Dojindo Laboratories, Kumamoto, Japan) and MTT assay. Cells (1 × 10^4^ cells/ml) were seeded in 96-well plates. After 24 h, the cells were pretreated with different concentrations of LPE (0.5, 5, 50 μg/ml) for 6 h, cotreated with 100 μM H_2_O_2_ for 24 h, incubated in CCK solution for 90 min at 37 °C incubator. Color development was measured at 450 nm using ELISA microplate reader. For MTT assay, 100 μl of 0.25 mg/ml MTT solution in PBS was added to each well. After incubation at 37 °C for 2 h, MTT solution was removed, and cells were lysed by solubilization solution (1:1 DMSO:ethanol). Color development was measured at 560 nm using ELISA microplate reader. To identify the molecule critical for the neuroprotective effects of LPE, cells were treated with inhibitor 30 min prior to treatment of LPE and H_2_O_2_.

### Preparation of cellular protein extraction and western blot analysis

Whole cell lysates were prepared using RIPA buffer (Millipore Corporation, Billerica, MA, USA) by adding protease inhibitor cocktail and phosphatase inhibitors (Roche Diagnostics, Basel, Switzerland). After washing cells twice with PBS, cells were harvested and collected by centrifugation at 12,000 rpm for 15 min. The pellets were resuspended in RIPA buffer. Protein concentrations were determined using bicinchoninic acid (BCA) assay kit with bovine serum albumin standard. Samples (30 μg protein per lane) were then separated in SDS-polyacrylamide gels and transferred electrophoretically to Immobilon-PSQ transfer membranes (Millipore Corporation, Billerica, MA, USA). Membranes were then placed immediately into a blocking solution (5 % nonfat milk) at room temperature for 30 min, and incubated with the following diluted primary antibodies; PARP, Caspase-3, p-p38, p38, β-actin (Cell Signaling technology, MA, USA) in TBS-T buffer (Tris–HCl based buffer containing 0.2 % Tween 20, pH 7.5) overnight at 4 °C. After washing (4 × 10 min), membranes were incubated with secondary antibody (monoclonal anti-mouse antibody or polyclonal anti-rabbit antibody (Santa Cruz Biotechnology, TX, USA) in TBS-T buffer at room temperature for 90 min. Horseradish-conjugated secondary antibody labeling was detected by enhanced chemiluminescence and blots were exposed to radiographic film. Pre-stained blue markers were used to determine molecular weights. Band intensities were measured using FluorChem™ SP software (Alpha. Innotech, San Leandro, CA, USA). Band intensities were normalized to β-actin or total p38.

### ROS production

ROS production was measured by fluorogenic dye 2’, 7’-dichlorodihydrofluorescein diacetate (H_2_-DCFDA), which is oxidized by intracellular ROS. Cells (5 × 10^3^ cells/well) were seeded in black 96-well plates and pretreated with vehicle or LPE for 6 h, treated with 50 μM H_2_-DCFDA for 30 min, and then cells were incubated with 100 μM H_2_O_2_ for 30 min. The cells were washed with PBS and fluorescent compound was detected by fluorescence microplate reader (SpectraMax i3, Molecular devices, CA, USA) with excitation and emission of 495 nm and 529 nm, respectively.

### Mitochondrial membrane potentials assay

Mitochondrial membrane potential depolarization, an early process in the apoptotic cell death, was measured using a cationic carbocyanine dye JC-1. JC-1 dye exists in the cytosol as a monomeric form (green) and also accumulated as a J-aggregates form (red) in the mitochondria. However, in apoptotic cells, JC-1 exists in monomeric form and stains the cytosol green. The monomers emit a green fluorescence (excitation and emission of 490 nm and 530 nm) and J-aggregates a red fluorescence (excitation and emission of 490 nm and 590 nm). Cells were seeded in confocal dish (coverglass-bottom dish). After pretreatment with vehicle or LPE for 6 h, cells was then cotreated with 100 μM H_2_O_2_ for 1 h. Cells were further incubated with JC-1 (chloride salt, Biotium, Hayward, CA, USA) staining solution (5 μg/ml) at 37 °C incubator for 15 min and rinsed with culture media. Mitochondrial membrane potential was estimated by measuring the fluorescence of free JC-1 monomers (green) to JC-1 aggregates in mitochondria (red) by FV10i FLUOVIEW Confocal Microscope (Olympus, Tokyo, Japan). Mitochondrial depolarization is indicated by increase in the proportion of cells emitting green fluorescence. To measure the red and green fluorescence intensity ratio, 1024 × 1024 pixels images were collected (*n* = 8). Red and green fluorescence intensity, respectively, in the individual cells were quantified using FV10i software (Olympus).

### Flow cytometry analysis

FITC Annexin V Apoptosis Detection Kit I (BD Biosciences, CA, USA) was used to detect the cell death. In brief, after treatment with vehicle or H_2_O_2_, H_2_O_2_ + LPE for 24 h, cells were trypsinized and resuspended in binding buffer (0.1 M HEPES/NaOH pH 7.4, 1.4 M NaCl and 25 mM CaCl_2_). 5 μl of Annexin V-FITC and 5 μl of propidium iodide (PI) were added and incubated for 15 min at room temperature in the dark. Cells were analyzed using flow cytometry (FACSCalibur, Becton Dickinson, CA, USA).

### Statistical analysis

The statistical analysis of the differences between the vehicle, H_2_O_2_ and LPE-treated groups was determined by one-way analysis of the variance (ANOVA) with Dunnett’s test. The analyses were performed using GraphPad PRISM software® (GraphPad PRISM software Inc., Version 5.02, CA, USA). Results are expressed as means ± standard errors (SE), and p values of < 0.05 were considered as significant.

## Results

### Effects of LPE on H_2_O_2_-induced cell loss in SH-SY5Y cells

**Fig. 1:**
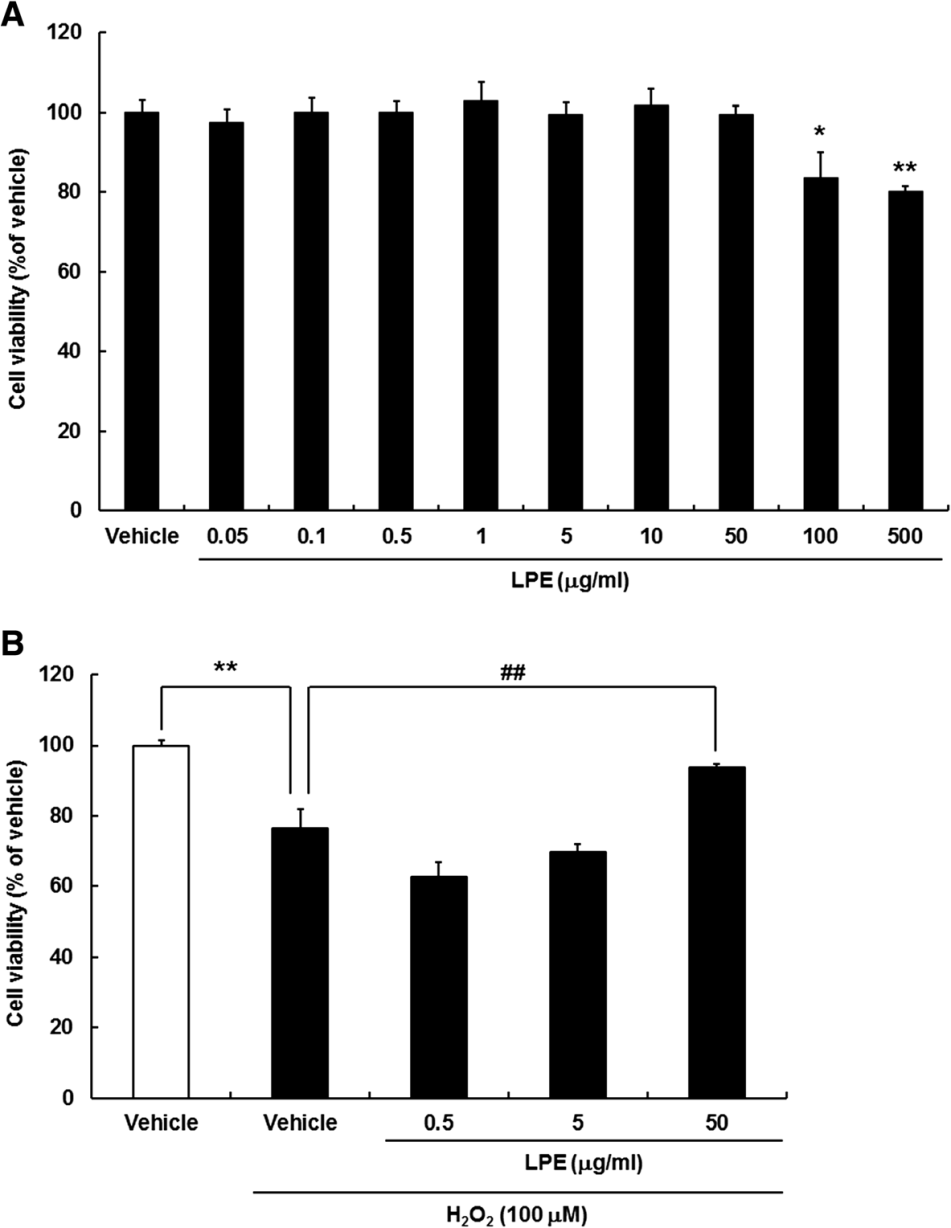
*Liriope platyphylla* extract (LPE) exerted neuroprotective effects against H_2_O_2_-induced cell loss in SH-SY5Y neuroblastoma cells. **a** SH-SY5Y cells were seeded in 96-well plates (5 × 10^4^ cells/ml) and cultured for 24 h. Cells were treated with the indicated concentrations of LPE for 24 h. LPE treatment at concentrations up to 50 μg/ml had no significant effect on SH-SY5Y cell viability. However, high LPE concentrations (100 – 500 μg/ml) decreased cell viability. The values shown are means  ±  standard errors (SE; *n*  =  8). *p < 0.05, **p < 0.01 compared to vehicle **b** Neuroprotective effect of LPE against H_2_O_2_-induced cytotoxicity in SH-SY5Y cells. Cells were pretreated with LPE for 6 h, and then co-treated with LPE and 100 μM H_2_O_2_ for 24 h. The values shown are means  ±  SE (*n*  =  8). **p < 0.01, compared to vehicle without H_2_O_2_, ^##^p < 0.01, compared to vehicle with H_2_O_2_

To determine the effects of LPE treatment on cell viability, SH-SY5Y cells were seeded in 96-well plates (5 × 10^4^ cells/ml), cultured for 24 h, and then treated with LPE at concentrations ranging from 0.05 to 500 μg/ml for 24 h. LPE treatment had no significant effect on SH-SY5Y cell viability at concentrations up to 50 μg/ml. High concentrations of LPE (100 μg/ml – 500 μg/ml) decreased cell viability (Fig. 1a). Therefore, we chose an LPE concentration range of 0.5 μg/ml – 50 μg/ml that did not induce cytotoxicity in SH-SY5Y cells. To assess its neuroprotective effects against H_2_O_2_, cells were pretreated with LPE for 6 h and then exposed to 100 μM H_2_O_2_ for 24 h. CCK analysis showed that H_2_O_2_ decreased SH-SY5Y cell viability, while 50 μg/ml LPE pretreatment conferred significant protection against H_2_O_2_-induced cell loss (Fig. 1b).

### LPE suppressed H_2_O_2_-induced intracellular oxidative stress and disruption of mitochondrial membrane potential (MMP)

**Fig. 2:**
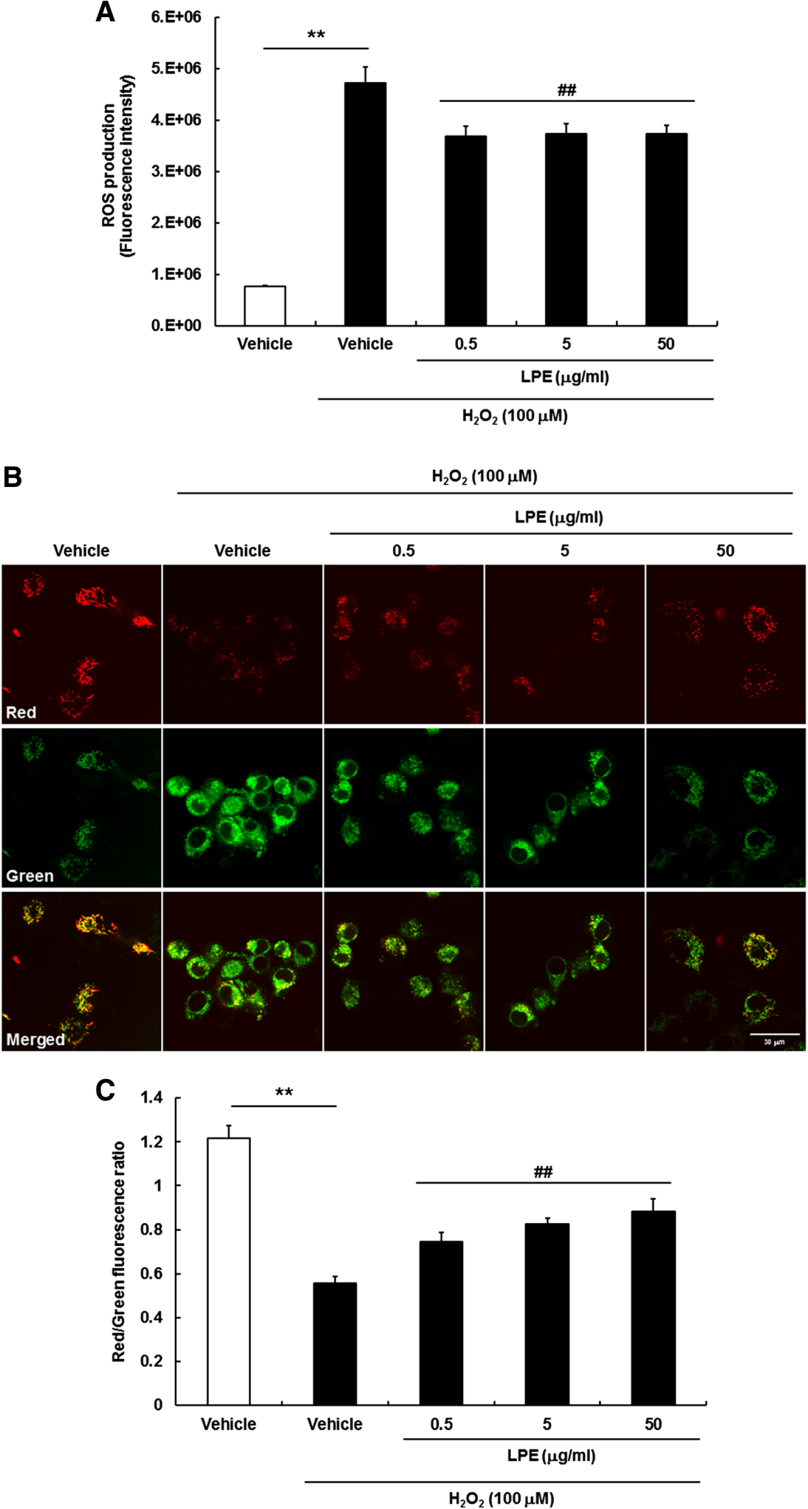
Effects of LPE on H_2_O_2_-induced oxidative stress and mitochondrial dysfunction in SH-SY5Y cells. **a** Total intracellular reactive oxygen species (ROS) levels were measured using the dichlorofluorescein diacetate (DCFDA) method. SH-SY5Y cells were exposed to LPE pretreatment for 6 h, and then labeled with 50 μM DCFDA for 30 min. Cells were then treated with 100 μM H_2_O_2_ and analyzed immediately using a fluorescent plate reader. Values are reported as means  ±  SE (*n*  =  8). **p  <  0.01, compared to vehicle without H_2_O_2_, ##p  <  0.01, compared to vehicle with H_2_O_2_ **b** Mitochondrial membrane potential (MMP) was assessed by confocal microscopy using JC-1 staining. Representative images showing red fluorescence (aggregated form) and green fluorescence (monomeric form). Cells were observed under 132× magnification. Scale bar  =  30 μm. **c** The graph shows the red/green fluorescence intensity ratio quantitative analysis. Values are reported as means  ±  SE (*n*  =  8). **p  <  0.01, compared to vehicle without H_2_O_2_, ^##^p  <  0.01, compared to vehicle with H_2_O_2_

Exogenously added H_2_O_2_ induced intracellular ROS generation and affected the accompanying cellular metabolic responses [19]. We evaluated the effects of LPE treatment on H_2_O_2_-induced endogenous ROS in SH-SY5Y cells. To measure the levels of intracellular ROS in H_2_O_2_-treated SH-SY5Y cells, we used the fluorescent dye H_2_-DCFDA, which is oxidized to fluorescent DCF by ROS. H_2_O_2_ treatment caused a marked increase of intracellular ROS generation; however, 0.5 μg/ml – 50 μg/ml LPE pretreatment significantly reduced ROS production (Fig. 2a). Therefore, LPE had an anti-oxidative effect on H_2_O_2_-induced oxidative stress.

ROS-mediated mitochondrial permeability transition pore (mPTP) opening causes mitochondrial dysfunction due to mitochondrial matrix swelling and outer membrane rupture. Therefore, we evaluated the effects of LPE on H_2_O_2_-induced disruption of MMP by JC-1 staining. Aggregated JC-1 exhibits red fluorescence in healthy mitochondria, while the monomeric form is characterized by green fluorescence when mitochondria are depolarized during apoptotic cell death. Intact mitochondria are indicated by an increase in the red/green fluorescence intensity ratio. In control cells (vehicle group), both red and green fluorescence were observed in healthy mitochondria, which were rod-shaped, in the cytoplasm (Fig. 2b and c). However, H_2_O_2_-treated cells showed decreased red fluorescence and increased green fluorescence, indicating loss of MMP. In addition, mitochondria in H_2_O_2_-treated cells were widely distributed in the cytoplasm (Fig. 2b and c). LPE pretreatment prevented the loss of MMP under H_2_O_2_-induced neurotoxic conditions. In particular, 50 μg/ml LPE pretreatment resulted in a JC-1 distribution similar to the vehicle group. These results suggest that the neuroprotective effects of LPE against H_2_O_2_-induced cell injury occurred via inhibition of MMP loss and oxidative stress.

### LPE prevented H_2_O_2_-induced apoptotic cell death in SH-SY5Y cells

**Fig. 3:**
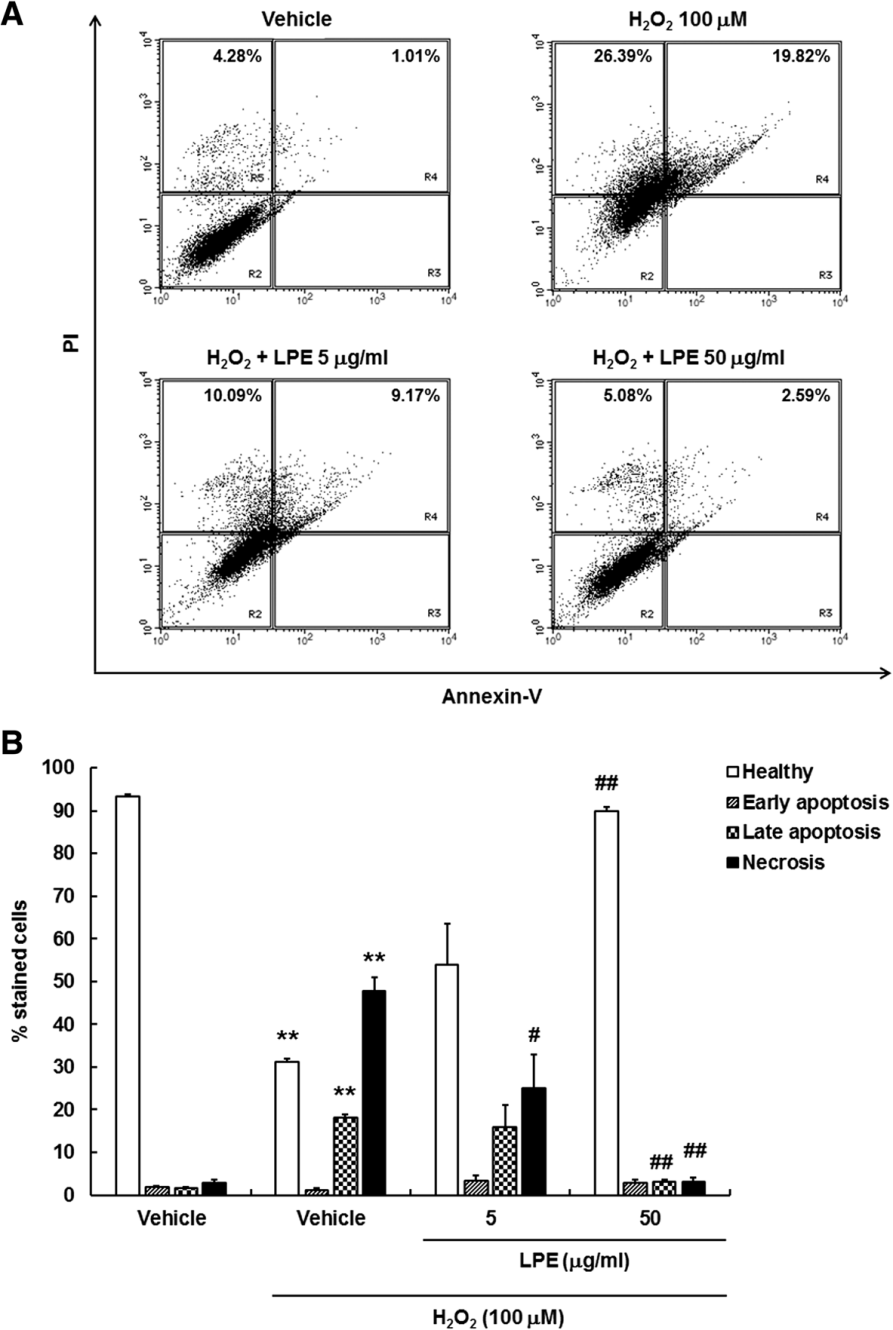
Effects of LPE on H_2_O_2_-induced cell death in SH-SY5Y cells. **a** Cells treated with vehicle, H_2_O_2_ or H_2_O_2_ + LPE (5 or 50 μg/ml) for 24 h were subjected to flow cytometry analysis after PI and Annexin-V staining. H_2_O_2_ treatment alone increased late apoptotic cells (R4, upper right quadrant) and necrotic cells (R5, upper left quadrant), but pretreatment of LPE protected against H_2_O_2_-induced cell death. **b** Quantitative data showed that percentage of healthy, early apoptotic, late apoptotic, and necrotic cells according to treatment. Values are reported as means  ±  SE (*n*  =  3). **p  <  0.01, compared to vehicle without H_2_O_2_, ^##^p  <  0.01, compared to vehicle with H_2_O_2_

**Fig. 4:**
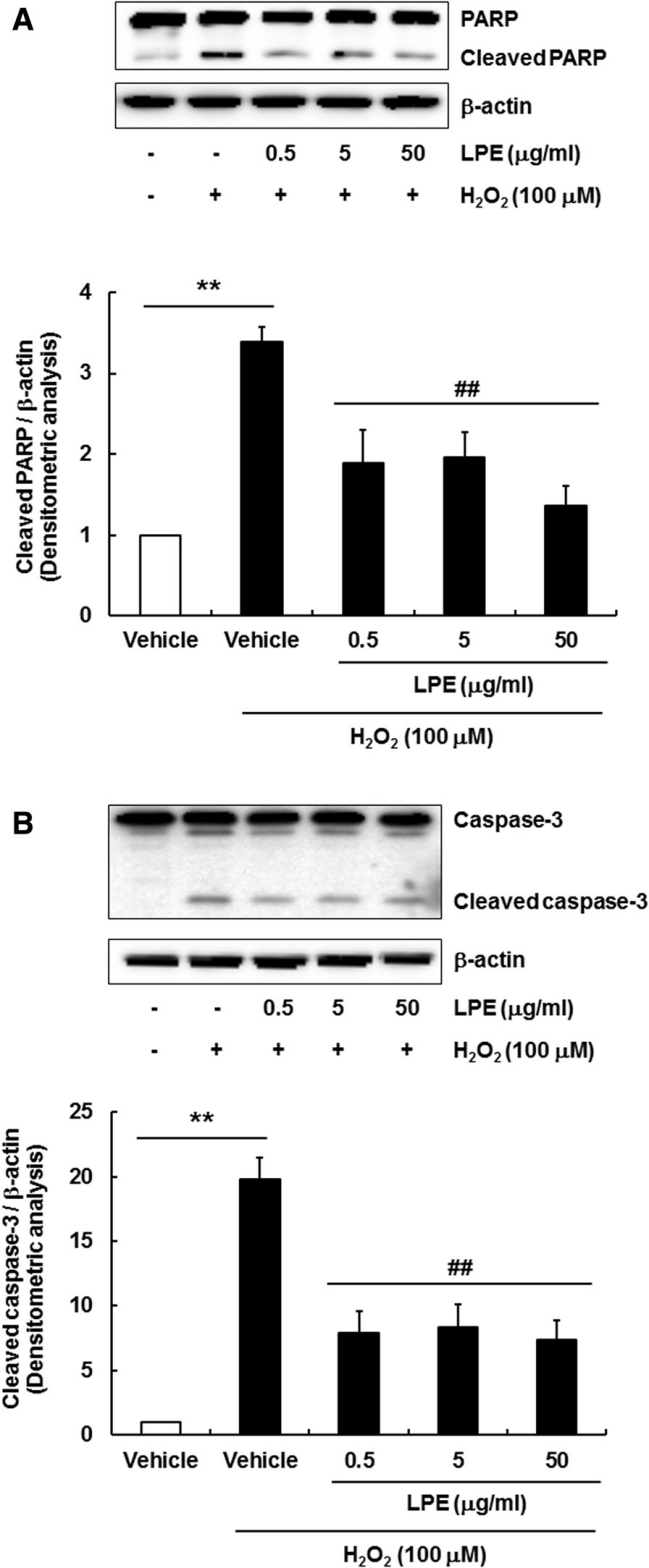
LPE attenuates H_2_O_2_-induced cleavage of PARP and caspase-3 in SH-SY5Y cells. Cells were pretreated with LPE for 6 h, and then co-treated with LPE and 100 μM H_2_O_2_ for 24 h. PARP **a** and caspase-3 **b** were examined using western blotting. H_2_O_2_ treatment alone resulted in PARP and caspase-3 cleavage, indicating that H_2_O_2_ induces apoptotic cell death in SH-SY5Y cells. LPE pretreatment protected against H_2_O_2_-induced cleavage of PARP and caspase-3. β-Actin was used as a protein loading control. A representative blot is shown from three independent experiments that yielded similar results. Quantification of band densities for cleaved PARP and caspase-3 were measured. Data are expressed as means  ±  SE (*n*  =  3). **p  <  0.01, compared to vehicle without H_2_O_2_, ##p  <  0.01, compared to vehicle with H_2_O_2_

H_2_O_2_-induced ROS and mitochondrial dysfunction were reported to induce apoptotic cell death in various organs, resulting in physiological and pathological changes [20, 21]. As apoptotic cell death is closely associated with the breakdown of MMP, we examined whether LPE protected SH-SY5Y cells against H_2_O_2_-induced cell death. After treatment with H_2_O_2_ and/or LPE for 24 h, cells were stained to Annexin-V and PI for flow cytometry. Cells are represented in dot plot as healthy (Annexin-V^−^/PI^−^, lower left quadrant), early apoptosis (Annexin-V^+^/PI^−^, lower right quadrant), late apoptosis (Annexin-V^+^/PI^+^, upper right quadrant) and necrosis (Annexin-V^−^/PI^+^, upper left quadrant. The percentage of late apoptotic and necrotic cells increased in H_2_O_2_ treatment, indicating that both apoptosis and necrosis are major events involved in H_2_O_2_-induced cytotoxicity in SH-SY5Y cells. LPE pretreatment remarkably reduced H_2_O_2_-induced cell death in late apoptosis and necrotic cell populations (Fig. 3a and b). Exposure to H_2_O_2_ alone increased apoptotic markers, such as poly(ADP-ribose) polymerase (PARP) cleavage and caspase-3 cleavage, in SH-SY5Y cells (Fig. 4a and b). LPE pretreatment effectively blocked PARP and caspase-3 cleavage. These results showed that LPE pretreatment attenuated the cleavage of PARP and caspase-3 due to H_2_O_2_ treatment (Fig. 4a and b). Therefore, LPE has anti-apoptotic properties against H_2_O_2_-induced apoptosis in SH-SY5Y cells.

### p38 MAPK modulation was involved in the neuroprotective mechanism of LPE against H_2_O_2_-induced cell injury

**Fig. 5:**
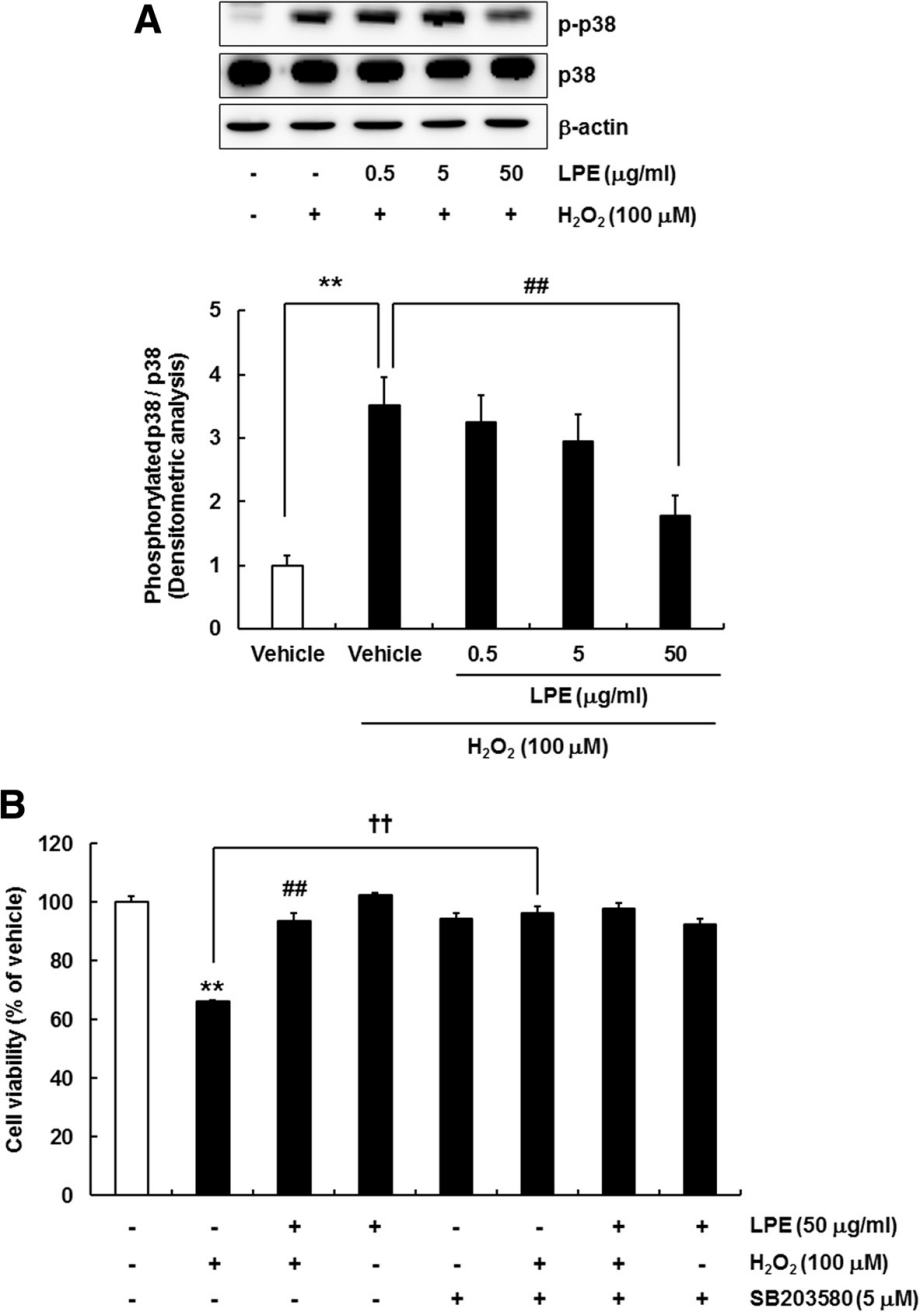
LPE exerts neuroprotective effects on H_2_O_2_-treated SH-SY5Y cells by modulating the p38 MAPK signaling pathway. **a** Cells were pretreated with LPE for 6 h, and then co-treated with LPE and 100 μM H_2_O_2_ for 24 h. Western blotting results demonstrated that H_2_O_2_ treatment activated phosphorylated (p)-p38; however, LPE pretreatment attenuated the p-p38 level. β-Actin was used as a protein loading control. A representative blot is shown from three independent experiments that yielded similar results. Quantification of band densities for phosphorylated p38/total p38 was measured. Data are expressed as means  ±  SE (*n*  =  3). **p  <  0.01, compared to vehicle without H_2_O_2_, ^##^p  <  0.01, compared to vehicle with H_2_O_2_ **b** Cells were pretreated with SB203580 (p38 MAPK inhibitor, 5 μM) for 30 min, 50 μg/ml LPE for 6 h, 100 μM H_2_O_2_ for 24 h, and then subjected to the 3-(4,5-dimethylthiazol-2-yl)-2,5-diphenyltetrazolium bromide (MTT) assay. Values are shown as means  ±  SE (*n*  =  8). **p  <  0.01, compared to vehicle without H_2_O_2_, ^##^p  <  0.01, compared to vehicle with H_2_O_2_, ††p  <  0.01, compared to H_2_O_2_ with inhibitor.

Previous studies suggested that ROS may activate the MAPK signaling pathway [22–25]. We investigated whether the MAPK signaling pathway, including extracellular signal-regulated kinase (ERK), c-Jun N-terminal kinase (JNK), and p38 MAP kinase, was involved in the neuroprotective effects of LPE against H_2_O_2_-induced cell injury. Specifically, SH-SY5Y cells were pretreated with LPE for 6 h, and then exposed to 100 μM H_2_O_2_ for 24 h. Activation of the MAPK signaling pathway was analyzed by western blotting. H_2_O_2_ treatment resulted in significant p38 phosphorylation; however, H_2_O_2_ treatment alone did not induce ERK or JNK activation (data not shown). Interestingly, we found that LPE only inhibited p38 phosphorylation in H_2_O_2_-treated SH-SY5Y cells (Fig. 5a). LPE did not affect the phosphorylation of ERK or JNK in H_2_O_2_-treated SH-SY5Y cells (data not shown). In addition, to determine whether the p38 signaling pathway was involved in the neuroprotective effects of LPE against H_2_O_2_-induced oxidative and toxic conditions, we used the p38 inhibitor, SB203580. Cells were seeded in 96-well plates (5 × 10^4^ cells/ml), cultured for 24 h, pretreated with SB203580 (5 μM) for 30 min, treated with LPE (50 μg/ml) for 6 h, and exposed to 100 μM H_2_O_2_ for 24 h. Cell viability was determined by MTT assay. H_2_O_2_ markedly decreased cell viability; however, LPE dramatically reversed the cell loss in the absence of SB203580 (Fig. 5b). In the H_2_O_2_ treatment group, cell viability was significantly improved in the presence of SB203580, indicating that the p38 signaling pathway mediated the H_2_O_2_-induced cell loss. No additional or synergistic protective effects were observed in cells co-treated with LPE and SB203580. These results indicated that LPE had neuroprotective effects against H_2_O_2_-induced cytotoxicity via attenuation of p38 phosphorylation.

## Discussion

LP is a traditional herbal medicine in Asian countries and has been used as a therapeutic drug for treatment of cough, inflammation, airway inflammation, obesity, and diabetes [12–16]. Furthermore, previous studies suggested that LP activated the neurogenic signaling pathway, and ameliorated the symptoms of neurodegenerative disease. LP prevented Aβ_1–42_ peptide deposition and increased NGF production in Tg2576 mice [17, 18]. Previous reports evaluated the relationship between the active compounds in LP and neuronal differentiation/brain-derived neurotrophic factor (BDNF)-mediated memory. Manufactured red LP increased NGF secretion and ERK phosphorylation in the B35 neuronal cell line and in PC12 cells [26]. Previous studies demonstrated that LP contains several active compounds, including spicatoside A, ophiopogonin A – D, and methylophiopogonanone A, B. Spicatoside A exerts its neurogenic effects by inducing neurite outgrowth and activating ERK and the phosphatidylinositol 3-kinase (PI3K)/Akt signaling pathway in PC12 cells [27]. Memory consolidation via upregulation of hippocampal BDNF levels was enhanced in spicatoside A-treated mice [28]. Ophiopogonin D has antioxidant and anti-inflammatory effects, and inhibits autophagic cell death in cardiomyocytes and human umbilical vein endothelial cells (HUVECs) [29–31]. Previous studies revealed that methylophiopogonanone B inhibited melanocyte dendrite elongation and induced actin cytoskeletal reorganization via Rho activation in normal human epidermal melanocytes and HeLa cells [32, 33]. However, there have been no previous reports regarding the neuroprotective effects of LP against H_2_O_2_-induced cell injury. The results of the present study indicated that LP ethanol extract had neuroprotective and anti-apoptotic effects against H_2_O_2_-induced cell injury via attenuation of mitochondrial dysfunction and intracellular oxidative stress, as well as p38 phosphorylation in human neuroblastoma SH-SY5Y cells.

Human neuroblastoma SH-SY5Y cells are widely used as an in vitro model in neuroscience research, including studies of neurobiology, neuronal differentiation, and neuroprotective events. Interestingly, SH-SY5Y cells differentiate into functional and mature neurons following RA treatment. Exogenous H_2_O_2_ treatment has been used to induce oxidative stress, because oxidative stress is related to neurodegenerative diseases and aging [34–36]. Exogenous H_2_O_2_ inhibited cell growth in various cell lines [37–40]. To determine the final concentration of H_2_O_2_, SH-SY5Y cells were treated with H_2_O_2_ at concentrations ranging from 10 μM to 500 μM for 24 h (data not shown). The cell viability was not affected at 10 or 50 μM H_2_O_2_, however, up to this concentration, it was decreased a concentration-dependent manner, with significant cytotoxicity being observed at concentrations > 250 μM. We thought that up to 250 μM was too high to evaluate the neuroprotective effects of LPE because only 20 − 50 % cells were survived in SH-SY5Y cells treated with up to 250 μM. Therefore, we used 100 μM H_2_O_2_ (20 − 30 % inhibition) to examine the neuroprotective effects of LPE in SH-SY5Y cells. Our data determined that LPE pretreatment prevented H_2_O_2_-induced cell loss and morphological changes, where 100 μM H_2_O_2_ decreased cell viability. Therefore, LPE had a neuroprotective effect on H_2_O_2_-treated SH-SY5Y cells.

Oxidative stress may affect cell proliferation, differentiation, and survival by activating signaling pathways. However, prolonged or high levels of oxidative stress cause neurotoxicity and neuronal cell death in neurodegenerative diseases and aging [41–43]. It is well known that H_2_O_2_-induced oxidative stress disrupts MMP and results in mitochondrial dysfunction [41–44]. In the present study, our data confirmed that H_2_O_2_ markedly increased both the intracellular ROS levels and mitochondrial dysfunction, and LPE pretreatment restored the ROS levels and MMP in H_2_O_2_-treated SH-SY5Y cells. Moreover, we examined whether H_2_O_2_-induced oxidative stress resulted in cell death. We confirmed that H_2_O_2_ treatment induced cell death by cleavage of caspase-3/PARP and Annexin-V^+^/PI^+^ staining. However, we found that LPE pretreatment reduced the cell death in SH-SY5Y cells. These results suggested that LPE acted as an anti-apoptotic agent by modulating ROS and mitochondrial function under H_2_O_2_-induced neurotoxic conditions.

Previous papers reported that H_2_O_2_ had cytotoxic effects in various in vitro model by a mechanism involving pro-apoptotic factors (Bax, caspases, PARP) activation and intracellular signaling pathway [45–51]. Especially, MAPK signaling pathway has been suggested as an important mechanism in oxidative stress-mediated neurodegenerative diseases [52, 53]. To address the possible mechanism of LPE-mediated neuroprotective effects, we determined whether LPE inhibited the activation of MAPK signaling pathway (ERK, JNK, p38) in H_2_O_2_-treated SH-SY5Y cells. As showed our result, H_2_O_2_ only activated p38 (Fig. 5a) not ERK and JNK (data not shown). In addition, p38 inhibitor blocked the cell loss in H_2_O_2_-treated SH-SY5Y cells; therefore, H_2_O_2_ required p38 activation for the induction of cytotoxicity. Our results demonstrated that LPE protected the cell growth against H_2_O_2_-induced cytotoxicity through inhibition of p38 phosphorylation. In addition, LPE only treatment did not affect the level of phosphorylated p38 in SH-SY5Y cells (data not shown). Therefore, we conjectured that LPE has neuroprotective effects by inhibition of p38 phosphorylation in a background of stressful state by H_2_O_2_-induced oxidative stress and apoptotic/necrotic cell death. Our results indicated that H_2_O_2_-induced p38 phosphorylation was sustained for over 120 min. We found that LPE suppressed p38 phosphorylation in H_2_O_2_-treated SH-SY5Y cells. These results demonstrated that p38 activation was required for H_2_O_2_-induced cell loss. In conclusion, LPE had anti-oxidant and anti-apoptotic effects via p38 MAPK downregulation.

## Conclusions

Collectively, the findings of this study indicated that LPE ameliorated H_2_O_2_-induced cell injury in SH-SY5Y cells. The protective effects of LPE were due to its ability to modulate ROS levels, apoptosis-related markers, and mitochondrial dysfunction via p38 MAPK regulation. The present study suggests that LPE may be a neuroprotective agent that can be used to prevent neurodegenerative diseases and brain aging.

## Abbreviations

LP: *Liriope platyphylla*
LPE: *Liriope platyphylla* extract
H_2_O_2_: Hydrogen peroxide
ROS: Reactive oxygen species
MMP: Mitochondrial membrane potential
PARP: Poly(ADP ribose) polymerase
